# Effect of Acupressure at P6 on Nausea and Vomiting in Women with Hyperemesis Gravidarum: A Randomized Controlled Trial

**DOI:** 10.3390/ijerph191710886

**Published:** 2022-09-01

**Authors:** Nor Azila Mohd Nafiah, Wei Keong Chieng, Ani Amelia Zainuddin, Kah Teik Chew, Aida Kalok, Muhammad Azrai Abu, Beng Kwang Ng, Nor Azlin Mohamed Ismail, Abdul Ghani Nur Azurah

**Affiliations:** Department of Obstetrics and Gynaecology, Universiti Kebangsaan Malaysia Medical Centre, Kuala Lumpur 56000, Malaysia

**Keywords:** morning sickness, pregnancy complications, antiemetics, urine ketone, acupressure wristband, normal intrauterine pregnancy, efficacy

## Abstract

Hyperemesis gravidarum is characterized by severe nausea and vomiting. This study aims to illustrate the efficacy of acupressure at P6 in treating nausea and vomiting in hyperemesis gravidarum. This parallel randomized controlled trial was conducted from 2016–2017 in a tertiary hospital. Hospitalized women with ≤16 weeks of gestation and moderate to severe nausea and vomiting classified using a modified PUQE score were randomly assigned in a 1:1 ratio to either apply an acupressure wristband at the P6 point three times daily or to receive regular doses of intravenous antiemetics. The primary outcome was differences in modified PUQE scores among the groups. The secondary outcomes were differences in the rate of urine ketone clearance and the frequency of requiring rescue antiemetics. Ninety women were equally randomized into two groups, with no dropout. There was a statistically significant difference in the degrees of nausea and vomiting between the groups at 8, 16, and 24 hours post-admission (p^8hours^= 0.001, p^16hours^ = 0.006, and p^24hours^ = 0.001). The requirement of antiemetics and the rate of urine ketone clearance between the two groups were also statistically significant, at *p* = 0.001 and *p* = 0.02 respectively. There were no side effects in either group. The P6 acupressure was efficacious in alleviating nausea and vomiting among hyperemesis gravidarum women. The trial was retrospectively registered on ClinicalTrials.gov (NCT05175079).

## 1. Introduction

Morning sickness is essentially nausea and vomiting in pregnancy (NVP) that affects approximately 70 percent of pregnant women worldwide [1]. It is a mild and self-limiting condition that can be controlled with conservative measures [2]. However, about 3.6 percent of pregnant women experience a more profound course of NVP known as hyperemesis gravidarum [3]. Hyperemesis gravidarum (HG) is diagnosed upon presentations of protracted NVP with the triad of more than 5% pre-pregnancy weight loss, dehydration, and electrolyte imbalance in the absence of other attributable medical conditions [4]. Recently, an updated definition of HG was proposed in which HG was defined as a condition that starts early in pregnancy before a gestational age of 16 weeks and is characterized by severe nausea or vomiting, an inability to eat or drink normally, and strong limitations in daily activities. Signs of dehydration are not mandatory according to the new definition [3].Unlike morning sickness, hyperemesis gravidarum may cause negative implications on maternal and fetal health [5,6].

The therapy options have largely remained unchanged for the past several decades. The choices include parenteral antiemetic medications, electrolyte repletion, and nutritional support. The interventions are decided and adjusted according to the frequency and severity of the symptoms. With the support of primary care professionals, mild NVP (PUQE score of ≤6) can be self-managed in the community. Moderate NVP (PUQE score of 7–12) may respond to complementary therapy, but antiemetics should be provided in cases without improvement. Severe nausea and vomiting and HG (PUQE score of >13) generally needs either ambulatory or inpatient hospital care to provide fluid and nutritional treatment. Lifestyle modifications such as small, frequent meals and avoidance of spicy and oily food may provide some symptom relief [7]. 

Antihistamines such as meclizine and promethazine have been used to treat NVP for decades. The neurotransmitter blockages, such as dopamine receptor antagonist (metoclopramide) and 5-HT serotonin receptor antagonist (ondansetron), used are often associated with side effects such as drowsiness and extrapyramidal symptoms [2,7]. Corticosteroids are reserved for patients with severe or refractory HG; however, the efficacy is contradictory [7]. In contrast, acupressure has the advantages of no side effects or drug interactions. It is noninvasive, convenient, and simple to apply, and it has a high degree of patient acceptance. Furthermore, it is more cost-effective [7].

The application of acupressure at the P6 (Nei Guan) meridian point is known to treat vomiting and other stomach problems in the practice of traditional Chinese medicine. Dundee et al. first revealed that acupuncture or acupressure at the P6 meridian point was as effective as the standard antiemetic in treating nausea and vomiting [8,9,10]. P6 (Nei Guan) is the sixth meridian point in the pericardium channel of Hand Jueyin, which is located on the anterior surface of the forearm about 2 inches proximal to the distal wrist crease between the tendons of the musculus flexor carpi radialis and musculus palmaris longus [11,12,13].

Acupressure and acupuncture are generally similar, except that the latter requires the use of needles. The special composition of blood vessels, mast cells, and nerve fibers at acupoints enables them to be easily activated for the mediation of acupuncture signals [14]. The mechanism of acupressure at the P6 point to prevent nausea and vomiting is not yet fully understood. It was found that acupuncture stimulated the release of β endorphins into the cerebrospinal fluid [15], thereby increasing the endogenous antiemetic tone [16]. The application of acupuncture has also been linked to the biological effects exerted by neurohumoral factors, neurotransmitters, and other chemical mediators in the nervous system for pain modulation [17]. 

The effectiveness of P6 acupressure on nausea and vomiting has been demonstrated in numerous conditions. It has been applied for pain and vomiting control postcraniotomy [18,19], for its antiemesis effect in postgynecological surgery [20], and for improvement of nausea and vomiting in pregnant women [21,22,23]. However, to date, there are no data comparing the antiemetic effect of P6 acupressure with conventional antiemetic drugs. Hence, this study was conducted to test the hypothesis that less nausea and vomiting would be experienced by patients wearing an acupressure band at the P6 point compared to those in a control group who received regular antiemetics.

## 2. Materials and Methods

### 2.1. Trial Design and Participants

This was a parallel, randomized controlled trial conducted in the Department of Obstetrics and Gynecology, Universiti Kebangsaan Malaysia Medical Center, from October 2016 through August 2017. This trial was approved by the Medical Research and Ethics Committee, Universiti Kebangsaan Malaysia Medical Center (Research Code: FF-2017-195), and was retrospectively registered at ClinicalTrials.gov (NCT05175079). Written consent was obtained from the participants prior to enrollment in the study. 

All pregnant women between the ages of 18 and 50 with normal intrauterine pregnancies of ≤16 weeks gestation presenting with severe nausea and vomiting to the emergency department were assessed by the medical officer, specialist, or consultant in charge for eligibility. The inclusion criteria included admission to the ward, onset of vomiting before 16 weeks, vomiting of at least 2 times per day, dehydration, loss of at least 5% of pre-pregnancy weight, ketonuria on admission, no urinary tract infection, and singleton pregnancy. The exclusion criteria were a nonviable pregnancy, a molar pregnancy, overt clinical features of thyrotoxicosis, a known case of medical illness associated with nausea and vomiting, multiple pregnancy, and patient refusal to participate. Non-hospitalized patients were not eligible to participate in the trial, as 8 hourly assessments were required; hence, they were treated according to local hospital protocol.

**Figure 1:**
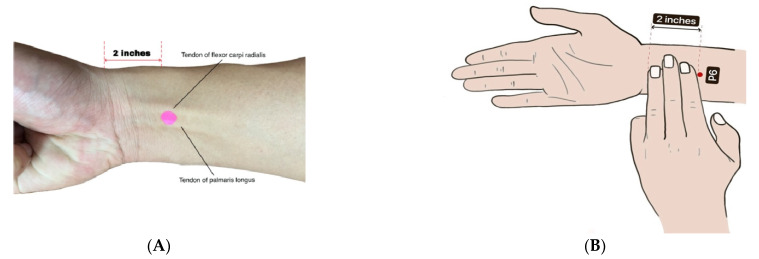
(**A**): The location of P6 acupressure point. The P6 point is indicated by pink dot. Red line represents the distance of 2 inches, which is proximal to the distal wrist crease. The tendons of flexor carpi radialis and tendon of palmaris longus are as labelled. (**B**) A schematic diagram showing the location of P6 point.

For the intervention group, acupressure wristbands were worn by the participants with the button of the wristband exerting pressure exactly on the P6 (Nei Guan) point (Figure 1A,B). One wristband was applied over the left forearm and one over the right forearm simultaneously. The application was 3 times per day for a duration of at least 10 min before breakfast, lunch, and dinner, respectively. Participants were instructed in proper determination of the P6 point and were observed during their first wearing of the wristband. The intervention was carried out for only one day during the participants’ admission to the wards. Intravenous fluids were given according to hospital protocol, and rescue antiemetic, intravenous metoclopramide at 10 mg, was given as needed if the vomiting was not reduced in amount. In the control group, intravenous fluids were given according to hospital protocol, and regular intravenous metoclopramide at 10 mg was given every 8 hours for 24 h. All participants from both groups were required to report any adverse events during the 24 h period to the trial staff.

### 2.2. Treatment Assignment

Information leaflets and explanation were given during recruitment. After giving their consent, the patients were enrolled and randomized to either the acupressure group or the control group. The allocation sequence was generated by a researcher who was not involved in statistical analysis using a randomly permuted blocks method (block sizes of 2, 4, and 6) on the www.randomization.com website, accessed on 1 June 2016 [24]. The allocation sequence was concealed until interventions were assigned. A matron and two staff nurses not involved in the trial helped to put either acupressure wristbands or antiemetics into containers of the same size before wrapping them with aluminum foil and sealing them in sequentially numbered brown-colored envelops. Blinding to participants, investigators, outcome assessors, and the statistician was not feasible, as there was no equivalent sham intervention available. However, no deviation from the protocol arose because of the trial context.

### 2.3. Sample Size

Based on Cohen’s formula 1988 [25] and considering the effect size X^2^ = 0.50, α = 0.05, and the power = 0.80, the appropriate number of samples was 39 in each of the two groups. The drop-out rate was estimated to be 10% given the nature of the trial, which required only a short duration of commitment from the hospitalized participants. Hence, a minimum of 86 participants was needed for this trial. A total of 90 were enrolled and equally distributed into two groups. 

### 2.4. Trial Outcomes

For the primary endpoint, a modified Pregnancy Unique Quantification of Emesis and Nausea (PUQE) score was used to objectively assess the degree of nausea and vomiting. It consisted of three questions than could be self-administered and required less than 2 min to be completed. Each question had a score range of 1 to 5. A score of <6 was categorized as mild, 7–12 was moderate, and >13 was a severe degree of nausea and vomiting. A baseline assessment of the modified PUQE score prior to administration of the intervention was conducted. Reassessments of the PUQE score were carried out at 8 h, 16 h, and 24 h postadmission. 

There were several secondary outcomes. The urine ketone level measured with a urine dipstick was documented during admission and after every 8 hours for 24 h. The time taken for urine ketone clearance was recorded. A clearance state was operationally defined as an undetectable ketone level using a urine dipstick. The frequency of antiemetic use over the same period was also recorded. The occurrence of any adverse event was also evaluated.

### 2.5. Statistical Analyses

Statistical analyses were performed using Statistical Package for Social Sciences (SPSS), version 23. A descriptive analysis was used to illustrate baseline characteristics of the study participants according to the assigned group. Continuous variables were reported as means and standard deviations, whereas categorical or ordinal variables were reported as absolute and relative frequencies. Pearson’s chi-squared test or independent *t*-tests were used to assess the primary and secondary outcomes. A *p*-value less than 0.05 was considered statistically significant. The intention-to-treat analytical method was adopted for all statistical analyses performed.

## 3. Results

**Figure 2:**
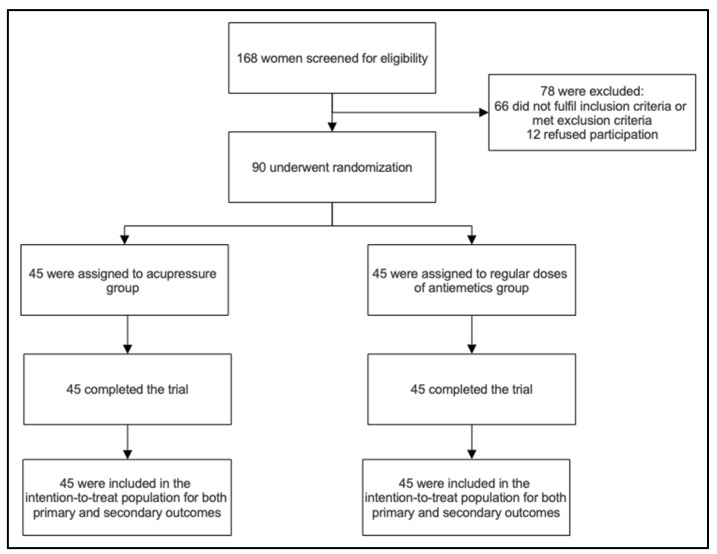
Enrollment and randomization flow chart.

A total of 168 pregnant women who presented to the emergency department with severe nausea and vomiting were screened for eligibility, of whom 102 were invited for enrolment in the study. A total of 90 pregnant women consented and were randomized into intervention and control groups with a 1:1 ratio. There were no dropouts or discontinuations of the intervention among the participants (Figure 2). There were also no side effects of either intervention reported by the participants. The baseline characteristics were balanced in both groups. Further details were as shown in Table 1. 

### 3.1. Primary Outcome

There was approximately two-thirds (64.4%) of the intention-to-treat population who experienced severe NVP, with the rest (35.6%) having moderate NVP. However, there was no statistically significant difference in the modified PUQE scores at baseline (on admission) between the acupressure and control groups (*p* = 0.378). 

After 8 h of admission, a significant difference (*p* = 0.001) in PUQE score was found between the two groups, with 73.3% showing moderate NVP and 26.7% achieving mild NVP in the acupressure group; in contrast, all of the 45 women in the control group had moderate NVP. 

Sixteen hours postadmission, there was a significant difference with a *p*-value of 0.006 between the two groups. In the acupressure group, almost two-thirds (60%) of the women had mild NVP, whereas only one-third (31.1%) attained the same in the control group.

After 24 h of admission, the PUQE scores still showed a significant difference between both groups with a *p*-value of 0.001. In the acupressure group, 93.3% showed a remarkable response to acupressure, with mild NVP scores, and only three women (6.7%) still experienced moderate NVP. Meanwhile, in control group, only 60% showed mild NVP. 

The results on the degrees of nausea and vomiting at admission, 8 h, 16 h, and 24 h are summarized in Table 2.

### 3.2. Secondary Outcomes

Upon admission, the intention-to-treat population had urine ketone ranging from 2+ to 4+ (Table 3). There was no significant difference found between the acupressure group and the control group, with 93.4% in the acupressure group and 80% in the control group having a urine ketone level of at least 3+. Postintervention, the acupressure group (21.73 ± 10.6) had a significantly higher rate of urine ketone clearance (*p* = 0.022) compared to the control group (27.73 ± 12.5), as shown in Table 4. 

For the frequency of antiemetics administered within 24 h, there was a significant difference between the two group (*p* = 0.001). In the control group, three doses of intravenous metoclopramide at 10 mg each were delivered according to protocol within 24 h. In comparison, in the acupressure group, intravenous metoclopramide at 10 mg was only given as necessary, and the results showed that 26.7% did not require any metoclopramide at all, 53.3% required a single dose, and the remaining 20% required only two doses. Further details are available in Table 5.

## 4. Discussion

The application of acupressure to the P6 point using wristbands for a cumulative of at least 30 min a day in pregnant women diagnosed with hyperemesis gravidarum was shown to significantly decrease the severity of nausea and vomiting more than regularly administered antiemetics when scoring was conducted at intervals of 8 hours (at 8 h, 16 h, and 24 h postadmission). Acupressure was also found to significantly reduce the need for antiemetic medications while increasing the rate of urine ketone clearance. Overall, the adherence to the usage of acupressure wristbands was perfect with no dropout.

Our findings echo a previous double-blinded trial that concluded a significant reduction in nausea, vomiting severity, and ketonuria when acupressure was used as an adjunct treatment to the existing standards of care for hyperemesis gravidarum in low-risk pregnancy [26]. A newer trial was also of the opinion that acupressure at the P6 point could apparently reduce the intensity of nausea and vomiting in pregnant women [27]. A recent network meta-analysis showed that acupressure was associated with better control of hyperemesis gravidarum symptoms than standard care and, at the same time, decreased the need for rescue antiemetics [28]. The quality of evidence in this meta-analysis was, however, very low. In contrast, a Cochrane review demonstrated insufficient high-quality evidence in ascertaining the efficacy of acupressure as an alternative to minimize the severity of nausea and vomiting in early gestation up to 20 weeks [29]. Another Cochrane review that exclusively included hyperemesis gravidarum patients reported an absence of trials that study the effects of P6 acupressure on nausea and vomiting in hyperemesis gravidarum patients [30]. Apart from recommending a consistent definition of hyperemesis gravidarum [30], two Cochrane reviews concomitantly highlighted the necessity to adopt standardized, justified outcome measurements [29,30]. 

The FDA has identified two adverse effects associated with acupressure devices, which are pain or discomfort at the region of application and skin irritation, while redness was also reported by a trial [26]. However, the usage of acupressure devices is generally safe since the FDA has recently stated its stance that general controls are sufficient to considerably assure the safety and effectiveness of acupressure devices [31], consistent with our findings that no participant reported any safety concerns or side effects. 

To the best of our knowledge, there is, to date, a limited number of trials on the efficacy of acupressure in hyperemesis gravidarum cohorts. A validated outcome measurement tool was employed to assess the primary endpoint of our trial. The no dropout in our trial conferred a higher statistical power to our findings. As acupressure application using wristbands is cost-effective, it can be easily replicated in the future. The implication of our trial was a contribution to the corpus of evidence on the effects of acupressure in alleviating hyperemesis gravidarum symptoms, which can encourage future meaningful syntheses of evidence to inform clinical decision. However, the robustness of our findings may be limited by the unfeasible blinding, as there was no availability of a reliable sham intervention. The effects of rescue antiemetics in the intervention group may overlap with the true effects of acupressure, resulting in the masking of nausea and vomiting symptoms and an inability to clearly delineate the attribution of the alleviation of symptoms between the two treatments. Future studies with outcomes at longer intervals can assess the longer-term sustainability of acupressure’s positive effects on nausea and vomiting in hyperemesis gravidarum. A standardized trial protocol in the future can underpin the influence of confounding factors, such as those that the time point and length of acupressure application exert on the efficacy of acupressure.

## 5. Conclusions

In conclusion, our study found a statistically significant effect of acupressure at the P6 point in reducing the degree of nausea and vomiting in women with hyperemesis gravidarum. The use of acupressure wristbands at the P6 point was also able to decrease the frequency of antiemetics and increase the rate of urine ketone clearance. The implication of our trial was the existence of an effective adjunct to alleviate the severity of nausea and vomiting in pregnant women with hyperemesis gravidarum, thereby improving their quality of life.

## Figures and Tables

**Table 1:** Baseline characteristics of participants.

Variable	All (*n* = 90)	Acupressure (*n* = 45)	Control (*n* = 45)	*p*-Value
**^a^** **Maternal age (years)**	30.9 ± 4.3	29.3 ± 4.5	30.8 ± 4.1	0.102
**^b^** **Ethnicity**				0.748
**Malay**	79 (87.8%)	39 (86.7%)	40 (88.9%)	-
**Chinese**	11 (12.2%)	6 (13.3%)	5 (11.1%)	-
**Indian**	-	-	-	-
**Other**	-	-	-	-
**^b^** **Parity**	-	-	-	0.084
**Primigravida**	40 (44.4%)	25 (55.6%)	15 (33.3%)	-
**Multipara**	50 (55.6%)	20 (44.4%)	30 (66.7%)	-
**^a^** **Gestational age (weeks)**	10.08 ± 2.5	10 ± 2.8	10.16 ±2.2	0.774
**^a^** **BMI (kg/m^2^)**	21.9 ± 3.7	21.6 ± 4.3	22.2 ± 2.9	0.464

^a^ Data expressed as mean ± SD; ^b^ data expressed as no. (%).

**Table 2:** Modified Pregnancy Unique Quantification of Emesis and Nausea (PUQE) scores in intention-to-treat population. * indicates statistically significant; *p* < 0.05.

	All (*n* = 90)	Acupressure (*n* = 45) no. (%)	Control (*n* = 45) no. (%)	*p*-Value
**PUQE score on admission**				0.378
**Mild NVP** **(<6)**	-	-	-	
**Moderate NVP** **(7–12)**	32 (35.6%)	18 (40%)	14 (31.1%)	
**Severe NVP** **(>13)**	58 (64.4%)	27 (60%)	31 (68.9%)	
**PUQE score at 8 h** **Mild NVP** **(<6)**	12 (13.3%)	12 (26.7%)	0 (0.0%)	0.001 *
**Moderate NVP** **(7–12)**	78 (86.7%)	33 (73.3%)	45 (100%)	-
**Severe NVP** **(>13)**	-	-	-	-
**PUQE score at 16 h**	-	-	-	0.006 *
**Mild NVP** **(<6)**	41 (45.6%)	27 (60%)	14 (31.1%)	-
**Moderate NVP** **(7–12)**	49 (54.4%)	18 (40%)	31 (68.9%)	-
**Severe NVP** **(>13)**	-	-	-	-
**PUQE score at 24 h**	-	-	-	0.001 *
**Mild NVP** **(<6)**	69 (76.7%)	42 (93.3%)	27 (60%)	-
**Moderate NVP** **(7–12)**	21 (23.3%)	3 (6.7%)	18 (40%)	-
**Severe NVP** **(>13)**	-	-	-	-

**Table 3:** Urine ketone levels on admission.

	All (*n* = 90)	Acupressure (*n* = 45) no. (%)	Control (*n* = 45) no. (%)	*p*-Value
**Urine ketone level**				0.160
**2+**	12 (13.3%)	3 (6.7%)	9 (20%)	
**3+**	54 (60%)	30 (66.7%)	24 (53.3%)	
**4+**	24 (26.7%)	12 (26.7%)	12 (26.7%)	

**Table 4:** Duration to achieve urine ketone clearance. * indicates statistically significant; *p* < 0.05.

	All (*n* = 90)	Acupressure (*n* = 45) mean ± SD	Control (*n* = 45) mean ± SD	*p*-Value
**Duration (hours)**				0.022 *
	24.73 ± 12.5	21.73 ± 10.6	27.73 ± 12.5	

**Table 5:** Frequency of antiemetic requirement in 24 h. * indicates statistically significant; *p* < 0.05.

	All (*n* = 90)	Acupressure (*n* = 45) no. (%)	Control (*n* = 45) no. (%)	*p*-Value
**Antiemetic dose**				0.001 *
**0**	12 (13.3%)	12 (26.7%)	0 (0.0%)	
**1**	24 (26.7%)	24 (53.3%)	0 (0.0%)	
**2**	9 (10.0%)	9 (20.0%)	0 (0.0%)	
**3**	45 (50.0%)	0 (0.0%)	45 (100.0%)	

## Data Availability

The datasets generated and analyzed during the current study are not publicly available to ensure the confidentiality of the trial participants but are available from the corresponding author on reasonable request.
